# Vascular Brain Damage in Thalassemia Syndrome: An Emerging Challenge

**DOI:** 10.22037/ijcn.v16i3.33794

**Published:** 2022-01-01

**Authors:** Mozhgan HASHEMIEH, Narjes JAFARI

**Affiliations:** 1 Pediatric hematologist and oncologist Imam Hossein Medical Center, Shahid Beheshti University of Medical Sciences, Tehran, Iran; 2 Pediatric Neurologist Imam Hossein Medical Center, Shahid Beheshti University of Medical Sciences, Tehran, Iran.

**Keywords:** Thalassemia, Hypercoagulable state, Thrombosis, Stroke, Infarct

## Abstract

Thalassemia syndromes are the most prevalent monogenic hemoglobinopathy in the world. In Iran, thalassemia is a public health problem because this country has been located on the thalassemia belt. In recent decades, considering that the life expectancy of patients with thalassemia has dramatically improved, some unrecognized complications have emerged in these individuals. One of these complications is a hypercoagulable state that may lead to thromboembolic events (TEE). The TEE may involve any organ in the body, including the central nervous system. Ischemic cerebrovascular events in thalassemic patients have been divided into two categories, namely overt stroke and silent cerebral infarcts (SCI). Overt stroke often develops in patients with beta-thalassemia major; however, patients with thalassemia intermedia usually suffer from SCI. This review article discusses brain vascular involvement.

## Introduction

Thalassemia syndromes are the most common hereditary hemoglobinopathy in the world. The hallmark of beta-thalassemia is the reduced (β^+^) or absence (β^o^) of β chain synthesis, which may lead to alpha chain excess. In β-thalassemia major (TM), there is the complete absence of β-chain production resulting in severe anemia in early infancy; however, in β-thalassemia intermedia (TI), due to decreased β-chain synthesis, clinical manifestations often appear later in childhood or even adolescence. In β-TI, the severity of symptoms may vary between mild manifestations of β-thalassemia minor and severe symptoms of TM (1). 

In Iran, thalassemia is a public health problem because this country has been located on the thalassemia belt. The average gene frequency rate in Iran is approximately 4%. The country-wide thalassemia prevention program was started in 1997 in Iran, resulting in a significant reduction in the thalassemic newborn rate (2). The main part of treatment in β-TM and some forms of β-TI is repeated regular transfusion resulting in iron accumulation in different vital organs. Therefore, these patients need iron chelator drugs to prevent hemosiderosis in those with transfusion-dependent β-thalassemia (TDT) (3). 

Magnetic resonance imaging (MRI) T2* is a highly sensitive technique for the assessment of organ-specific hemosiderosis (4). In recent decades, the life expectancy of patients with TDT has dramatically improved since some unrecognized complications have emerged in these individuals. Profound hemostatic alterations are among these morbidities which may occur in TM and especially TI patients (5). Multiple factors have been responsible for a hypercoagulable state in these patients (6-8). These thromboembolic events (TEE) may occur both in splenectomized and non-splenectomized patients (9). The TEE may involve various organs in the body, including deep vein thrombosis (DVT), portal vein thrombosis, pulmonary embolism, and even arterial occlusion (10,11). Furthermore, brain vascular involvement and cerebral micro-thrombosis are other complications that may occur in TDT and non-transfusion-dependent thalassemia (NTDT) (12).

**Mechanism of Hypercoagulability in β-Thalassemia**

Multiple abnormalities in platelets, red blood cells (RBC), coagulation factors, and physiologic anticoagulants, including protein C, protein S, and antithrombin III (ATIII), are responsible for the pathogenesis of a hypercoagulable state in thalassemia syndromes (13). As previously mentioned, TEE are more common in NTDT in comparison to those in well-transfused TDT. In a large epidemiological study performed on 8860 patients in the Mediterranean area and Iran, TEE were observed to be 4.38 times more prevalent in β-TI, compared to those in β-TM (14). In addition, splenectomized patients with NTDT are more vulnerable to episodes of thromboembolism (15). In NTDT patients who were splenectomized, a nucleated RBC count of > 300 × 10^6^/L, platelet of > 500 × 10^6^/L, and RBC transfusion naivety have been correlated with the increased incidence of TEE (16). 

Independent risk factors for the development of TEE in thalassemia syndromes include splenectomy, a serum ferritin level of > 1000 ng/ml, a hemoglobin level of < 9 gr/dl, and age of > 35 years (15). In patients with β-TM, the expression of P-selectin and CD63 have been increased. These two markers are the indicators of in vivo platelet activation resulting in increased platelet aggregation. In splenectomized patients, the platelet counts significantly increase; nevertheless, these platelets have a shorter life span due to the enhanced rate of platelet consumption (17). 

Moreover, RBC has a critical role in the pathophysiology of a hypercoagulable state in thalassemia. In these patients, the oxidation of globin subunits in RBC leads to the formation and then precipitation of hemichromes. These elements may cause the disintegration of heme and, finally, the release of toxic iron species. The free iron leads to the formation of phosphatidylserine, which is one of the RBC antigens. Phosphatidylserine causes deformation, rigidity, aggregation, and finally, premature death of RBC in circulation. Therefore, enhanced RBC aggregation and cohesiveness occur, and thrombin generation increases (18). 

Moreover, in patients with thalassemia, the levels of endothelial adhesion proteins (e.g., E–selection), intercellular adhesion molecule-1, von Willebrand factor, and vascular cell adhesion molecule-1 have been enhanced (13). One of the most important contributing factors in the pathogenesis of TEE in thalassemia syndromes is the reduction of protein C, protein S, and ATIII, all of which are natural physiologic anticoagulants (13, 19-21). In thalassemic patients, due to multiple reasons, such as hepatic hemosiderosis and viral infections, varying degrees of liver dysfunction occur, and proteins C and S are very sensitive even to mild hepatic dysfunction (22). 

In addition, in thalassemic patients, prothrombin fragment 1.2, which is a marker of thrombin generation, has an important role (23). Furthermore, splenectomy results in thrombocytosis enhanced platelet aggregation, and an increased number of damaged RBC. All the aforementioned parameters could increase the risk of thromboembolism (18).

**Consequences of Thromboembolism for Central Nervous System**

In patients with β-TM and β-TI, both arterial and venous TEE could be detected (14). Regarding the OPTIMAL CARE study, thromboembolic disorders (often venous thrombosis) were the fifth most prevalent morbidity among thalassemic patients occurring in almost 14% of patients (24). Recently, there has been a report on an increasing number of cerebrovascular episodes, such as overt stroke and silent cerebral infarcts (SCI), in patients with thalassemia and sickle cell anemia (25).

**Incidence of Brain Lesions**

Brain vascular involvement occurs in 29-83% of TI patients; however, the rate of asymptomatic brain lesions in healthy individuals is within the range of 0-11%; therefore, these lesions are more likely to be pathological rather than regular (26,27). In different studies, there is a wide range of TEE in patients with thalassemia (both TM and TI populations), within the ranges of 0.9-4% and 3.9-29%, respectively (25). 

Ischemic cerebrovascular events in thalassemic patients have been divided into two categories, namely overt stroke and SCI. In SCI, the patients are often asymptomatic, and the detection of lesions without brain imaging methods is not possible (28). In patients with β-TM, often overt stroke develops; however, patients with β-TI usually suffer from silent stroke (25). 

The presence of SCI on brain imaging can be related to an increased risk of subsequent overt stroke, a transient ischemic attack, and a marked reduction in cognitive function, both in children and adults (29). Moreover, in case of asymptomatic brain lesions in thalassemic patients, some authorities alter the therapeutic modalities; therefore, the early detection of these lesions has clinical importance (30). On the other hand, these asymptomatic brain lesions may cause permanent neurological deficits (31). 

Masood et al. reported that in patients with β-TM in the post-transfusion period, hypertension, seizure, headache, and intracerebral hemorrhage rarely occur (32). Furthermore, white matter lesions have a significant correlation with neurocognitive disorders leading to impairment in functional performance. The severity of neurodevelopmental disability relates to the number of white matter lesions (27). Regarding the risk of stroke due to cerebral thrombotic events, diagnostic brain MRI or magnetic resonance angiography (MRA) could be helpful to detect subclinical vascular damage in β-TI patients. Sometimes, microvascular damage may not be detected only by a single MRI in a period; therefore, it is recommended to repeat brain MRI at least every 2-3 years in high-risk groups, including splenectomized older patients with thrombocytosis (26).

**Characteristic of Central Nervous System Lesions**

In patients with β-TI, SCI have been diffusely reported in different regions of the hemispheres, but most commonly in parietal and frontal white matter. In the majority of patients, the subcortical white matter has been affected. It is also worth mentioning that TEE almost have a venous origin (33). Nemtsas et al. have reported that cerebrovascular involvement in β-TI patients, in comparison to that of β-TM patients, is often asymptomatic; however, in these patients, lesions have a smaller size and the involvement of cortex has not been reported mostly in deeper structures of the brain (25). Some patients with β-TI have numerous and multiple silent infarct lesions on imaging (up to 73 lesions) (34). 

Taher et al. reported that brain MRI could easily detect white matter lesions or brain atrophy due to ischemic events in splenectomized adults with TI. Moreover, the spectrum of white matter lesions may vary from mild perivascular changes to large areas with numerous small cavitation, variable loss of fibers, and arteriosclerosis. Additionally, irregular periventricular high signal intensity lesions are often indicators of microcytic and patchy infarcts in patients with β-TI (27).

**Pathophysiology of Brain Lesions**

Multiple factors have been proposed as the mechanisms of SCI in TI patients; nevertheless, the exact pathophysiology of ischemic cerebral infarcts has not been clarified. The majority of published articles evaluated the small samples of patients and mostly explored the nature of the disorder (27). Possible underlying etiologies include microangiopathy, venous thrombosis, and large vessel involvement (28); however, a hypercoagulable state is the most important contributing factor in the pathogenesis of SCI in β-TM and especially β-TI (6). 

Abnormal RBC in thalassemic patients have a pathological tendency to aggregation and consequently more thrombin generation and increased thromboembolic presentations. Therefore, regular transfusions may reduce TEE by decreasing damaged RBC (35). Advanced age is another risk factor correlated with the development of SCI in thalassemic patients; therefore, the mechanism of injury probably affects longitudinally, and numerous risk factors are required to accumulate for presenting these lesions (27). 

Furthermore, in splenectomized TI patients, TEE occurs more commonly due to the increased number of damaged RBCs remaining in the circulation. In addition, in splenectomized individuals, thrombogenesis renders the patients for thrombosis. Moreover, a high level of toxic iron species in non-transferrin bound iron (NTBI) is a significant contributing factor for the development of large arterial stenosis (27). The NTBI can cause oxidative vessel injury and may explain the pathophysiology of vascular damage in these patients (36).

**Diagnostic Modalities**

Brain MRI is a useful diagnostic method for the detection of asymptomatic or subclinical lesions in the central nervous system (CNS) of thalassemic patients. Karimi et al., in a retrospective analysis conducted on 30 splenectomized β-TI patients, observed pathological findings in the MRI of 28% of patients. Six patients had changes in white matter indicating ischemia, and two patients had evidence of small infarction. Karimi et al. concluded that splenectomized β-TI patients with a history of irregular transfusions and thrombocytosis (a platelet count of more than 500000/ml) have a major risk factor for developing vascular brain damage. In these patients, diagnostic MRI should be considered when they reach 20 years or even before to detect asymptomatic or subclinical vascular damage as soon as possible. Furthermore, Karimi et al. have recommended repeating brain MRI every 3-5 years afterward. A microvascular injury could not be assessed only by a single MRI study. Furthermore, further studies with different imaging techniques are needed to evaluate the extent of ischemic damage (31).

Cerebral venous sinus thrombosis (CVST) is another complication that may occur in thalassemic patients. The clinical presentations of this disorder may be life-threatening and lead to neurological deficits (37). Brain computed tomography scan is not a helpful diagnostic method for the detection of CVST (31). The best method for screening asymptomatic brain vascular damage in the early stages is diffusion-weighted magnetic resonance imaging (25). Unlike MRI, positron emission tomography-computed tomography (PET-CT) scan could not be helpful in the detection of SCI in patients with β-TI. The most substantial benefit of PET-CT is the identification of neuronal dysfunction, which is a common finding in thalassemia patients due to iron overload. Therefore, the combination of PET-CT and MRI could be a better option for the identification of stroke and functional neurological deficits in TI patients (33).

Another modality is MRA that can be used for the evaluation of large-vessel disease. Musallam et al. reported that although the large-vessel disease is a prevalent feature in splenectomized β-TI, it cannot explain the etiology of SCI in these patients. Musallam et al. concluded that the addition of MRA to MRI can detect a larger percentage of TI patients with silent neuroimaging deficits. These neuroimaging findings could be related to the occurrence of stroke in functional neurologic deficits in the future (29). Teli et al. and Ashjazadeh et al., in their studies, have shown that transcranial Doppler (TCD) ultrasound on TI patients shows that their intracranial circulation is higher than that of healthy controls (38,39). The TCD ultrasound, similar to MRA, can reveal large-vessel disease that does not seem to be associated with SCI (35).

**Biomarkers of Brain Ischemia**

Neuron-specific enolase (NSE) and S100 calcium-binding protein β (S100β) are two biomarkers that can be used for the identification of brain ischemia and CNS injury (40). In conditions associated with glial cells or neuronal damage, including ischemic stroke, brain hypoxia, or head trauma, the levels of NSE and S100β elevate. Kanavaki et al., in a study performed on 30 patients with NTDT, showed that the results of TCD correlate with the biomarkers for brain ischemia (28). 

**Treatment**

Stroke is the third leading cause of mortality (41). Cerebral TEE, both overt and silent, have been reported in thalassemia patients, especially in TI. The SCI may lead to brain damage and increase the risk of transient ischemic attack and major stroke in the future (34). To date, there have been no documented clear-cut guidelines regarding who might benefit from antiplatelet aggregation drugs before the appearance of overt neurological symptoms (41).

**Aspirin**

Aspirin can block thromboxane A_2_ synthesis and reduce platelet aggregation. Aspirin could diminish the conversion of arachidonic acid to thromboxane β_2_, causing platelet inactivation (42). Aspirin may have a protective effect on TEE in splenectomized β-TI patients with thrombocytosis (13). Taher et al. have shown a significant reduction in the recurrence rate of thrombosis after the first event in patients undergoing treatment with aspirin (17). 

Karimi et al., in a study conducted on 22 patients with β-TI and 13 patients with β-TM, compared white matter lesions between the first MRI and 3 years afterward. Karimi et al. divided patients into two groups according to aspirin consumption. The dose of aspirin was 80-100 mg once daily. The results showed that only one case developed new lesions or had an increase in the number of lesions, compared to 34.5% of cases not consuming aspirin. Moreover, in the aforementioned study, the difference was not significant. However, Karimi et al. recommended low-dose aspirin for high-risk thalassemic patients to prevent new lesions or progression of brain lesions. Patients with splenectomy, thrombocytosis, severe iron overload, and older age have been considered high-risk groups (41). The cut-off value of platelet count for the beginning of aspirin therapy (>500 × 10^9^/L versus >800-1000 × 10^9^/L) is controversial requiring further evaluation in future studies (35). 

**Blood Transfusion**

The mechanism of regular blood transfusion in the prevention and treatment of TEE in thalassemia patients is the reduction of phosphatidylserine exposure on the surface of RBC (43). Regular blood transfusions are recommended for patients with symptomatic CNS thrombosis (44).

**Direct Oral Anticoagulants**

Direct oral anticoagulants (DOACs) with direct inhibition of factor II a (dabigatran) or Xa (rivaroxaban and apixaban) have been used for stroke prevention in atrial fibrillation (45). These novel agents have multiple benefits over warfarin, such as few drug and food interactions, rapid onset of action, and predictable pharmacokinetics. Patients consuming DOACs often do not require regular coagulation monitoring (46). Despite the extensive use of DOACs in patients with atrial fibrillation and prevention of thrombotic episodes after the surgery, the experience of using these drugs in patients with thalassemia is very limited (13). 

Apostolou et al. reported the results of rivaroxaban usage in eight patients with hemoglobinopathy (four patients with sickle cell disease and four patients with β-TM). Five patients had nonvalvular atrial fibrillation and received rivaroxaban for the prevention of emboli and stroke (i.e., the primary prevention). For the other three cases, rivaroxaban was used for the treatment of DVT and the prevention of the recurrence of DVT and pulmonary embolism (i.e., secondary prophylaxis). The follow-up period was 6-34 months, in which none of the patients experienced any thrombotic event or bleeding (47). 

However, the most important toxicity of DOACs is hepatotoxicity, and thalassemic patients have liver dysfunction for many reasons. Iron accumulation, transfusion-transmitted infections, including hepatitis B virus and hepatitis C virus, and finally, a natural course of their disease may lead to liver damage in thalassemic patients. Therefore, in case of using DOACs for thalassemic patients, special attention is required, and regular monitoring of liver function tests is mandatory (48). The administration of rivaroxaban in thalassemic patients may be challenging, and further studies with larger samples are needed to ensure the safety of these drugs in thalassemia (47).

## In Conclusion

In recent years, the survival of patients with TDT has dramatically improved since brain vascular abnormalities, such as overt and silent stroke, have been emerged in these individuals. It seems that cerebral infarction and vascular injury begin in late childhood. In these patients, diagnostic MRI can be helpful to monitor early asymptomatic or subclinical vascular damage in the brain, especially when they reach the second decade of life. However, high-risk patients with thrombocytosis (a platelet count of >500,000 mm^3^), splenectomy, and severe iron overload should be under strict follow-up, be evaluated on a regular periodic basis, and undergo brain MRI at least once every 3 years. 

The emphasis on effective iron chelation, the addition of low-dose aspirin for the prevention of thromboembolic sequelae in high-risk thalassemia patients, and the inclusion of brain MRI in routine periodical evaluation are all advised for thalassemia patients to treat the individuals with asymptomatic brain damage as soon as possible.

## Author’s Contribution

Narjes jafari was corresponding author and wrote the section of Neurology in this manuscript. Mozhgan Hashemieh designed this research and wrote the section of hematology in the manuscript.

## Conflict of interest

The author has no conflict of interest with the subject matter of the present study.
